# Innovative Strategies for Talent Cultivation in New Ventures Under Higher Education

**DOI:** 10.3389/fpsyg.2022.843434

**Published:** 2022-03-30

**Authors:** Shiyan Liao, Chunhui Zhao, Mengzhu Chen, Jing Yuan, Ping Zhou

**Affiliations:** ^1^Teaching Evaluation and Educational Inspector Center, Zhaoqing University, Zhaoqing, China; ^2^Hebei Agricultural University, Baoding, China; ^3^School of Finance and Economics, Hebei Normal University of Science and Technology, Qinhuangdao, China; ^4^School of Economics and Management, Wuhan University, Wuhan, China; ^5^School of Foreign Studies, Hunan University of Humanities, Science and Technology, Loudi, China

**Keywords:** cultivation of innovative talents, higher education, talent capital, technological innovation, innovation performance

## Abstract

This study aims to help enterprises enhance their innovation capabilities in the environment of knowledge economy globalization and stand out in the fierce industry competition. Firstly, data on existing higher education theories and innovation theories are analyzed. Secondly, two companies in the sample data are selected for detailed analysis. Finally, research conclusion and corresponding talent management strategies are presented. The results show that the cumulative contribution value of employees is 87.496%. The cumulative contribution value of human capital is 70.322%. The contribution value of cumulative innovation performance is 61.658%. The cumulative contribution value of R&D investment is 45.306%. The coefficient for the overall sample size is 0.509. The employee quality coefficient is 0.452. The correlation coefficient for educational attainment is 0.598. The high-tech service industry has the highest correlation coefficient at 0.504. The auto industry has the highest coefficient at 0.669. The experimental research has drawn the following conclusion: (1) Talents positively affect enterprise innovation performance; (2) Research and Development (R&D) investment has a positive correlation with enterprise innovation performance; (3) R&D investment has a positive correlation with talents. Through experimental research, the education level of employees is measured by academic qualifications, but the essence of academic qualification measurement is the level of knowledge and skills that employees have. In summary, the study can extend the strategic analysis of the cultivation of innovative talents and play a valuable auxiliary role in cultivating innovative talents.

## Introduction

The problem of the shortage of high-quality technical and skilled personnel in China’s industrial upgrading and economic restructuring has become increasingly prominent (Peng et al., 2020; Sun et al., 2020). Therefore, vigorously cultivating technical and technical talents that are compatible with the upgrading of industrial structure and social and economic development, and improving the modern higher education system have become an urgent problem (Sowmiya and Kalaiselvi, 2020; Sun et al., 2020). Higher education institutions should adapt to the new needs of economic and social development for the level and specifications of technical and technical talents. Higher education institutions should explore the innovation of vocational education talent training models, promote the connection of higher education and enterprise innovative talent training, and lay the foundation for the development of an innovative talent training system (Peng et al., 2020; Zeng and Tang, 2020; Wang et al., 2021).

Qian et al. (2018) found that empowered leaders were positively correlated with followers’ feedback seeking. Employee feedback seeking is positively related to task performance, person in charge and voice. Employee feedback (Aleksandr and Olga, 2019) seeking mediates the positive relationship between empowered leadership and task performance, the person in charge, and the right to speak. This shows that cultivating innovative enterprise talents also needs to pay attention to the intermediary role of enterprise leaders. Chen (2019) stated that the challenges faced by foreign employees come from the assigned tasks, unknown environment (Dorrer et al., 2018), language barriers and cultural differences. Excessive pressure can cause ideological and psychological burdens on expatriates, and even cause physical symptoms (Wu and Wu, 2017; Wu et al., 2019; Wu and Song, 2019). However, proper pressure can play a leading role and promote the smooth progress of work (Zheng et al., 2018; Yuan and Wu, 2020). It is necessary to pay attention to the pressure diversion and balance of talents while cultivating new ventures (Ye and Chen, 2021).

Therefore, it is unique in that it is based on the theory of innovative talent training. From higher education, discuss the impact of talents on innovation. Starting from the data, find effective influencing factors. Because of the influencing factors, reasonable and effective strategies for training innovative talents have been explored for the practice of enterprise innovation management. Organization structure: Section 1 is the Introduction. This is an analysis of the current situation of new talent training, which leads to the vital analysis direction of this research. Section 2 is the Method. The latest theories on the cultivation of innovative talents are introduced. Statistical software is used for descriptive statistical analysis. Section 3 is the Result. Various influencing factors of innovative talent cultivation are analyzed. Section 4 is the Discussion. The utility of the obtained results is analyzed. Section 5 is the Conclusion. Results are summarized and research gaps and future prospects are presented.

### Literature Review

Rogoza et al. (2018) believed that fragile self-esteem and admiration exhibited contradictory relationship patterns with shyness and loneliness, while competition heralded low empathy. Thought that the two satisfaction factors of trust and profit could be regarded as the specific satisfaction of online entrepreneurial groups, especially the trust factor. It is more worthy of attention in the further research of social media online entrepreneurship courses. Used Tianjin University’s MBA students as a sample to analyse the relationship between the dark triad, entrepreneurial self-efficacy and EI. The results show that the dark triad positively predicts EI, and ESE partially mediates the dark triad and EI. Narcissism or psychosis harms ESE and EI. Narcissism or psychosis has a non-linear effect on EI. Machiavellianism has a positive impact on ESE and EI. ESE has an intermediary effect on the three members of the Dark Triad and EI. Eden’s mobile-accessible transportation service platform, Wu W. et al. (2020) proposed a social business model for the disabled. This social business model illustrates how information and communication technologies (Kostin et al., 2019; Wei, 2019) can be combined with transportation service providers and government resources to meet the transportation needs of people with disabilities. This shows that the training of talents for new ventures should also focus on training social business models (Artaphon and Pallop, 2019).

From the analysis of the literature results, the cultivation of innovative talents pays excellent attention to analyzing the factors affecting the cultivation of creative skills. Only by adapting to local conditions can the efficiency of talent cultivation be improved (Feng and Chen, 2020; Wu Y. J. et al., 2020).

### Relevant Theories and Research Methods of Talent Training for New Ventures in Higher Education

#### Higher Education Theory

In China, higher education is a variety of academic and professional education conducted since completing secondary education. Its connotation has three levels (Sloam et al., 2021).

1. The starting point for the graduation level of secondary education is the basic measure of higher education.
2. If it is academic and professional education above the graduation level of secondary education, regardless of its form, it belongs to the conceptual category of higher education.
3. The task of higher education is to train senior talents with innovative spirit and practical ability, develop science, technology, and culture, and promote socialist modernization.

**FIGURE 1:**
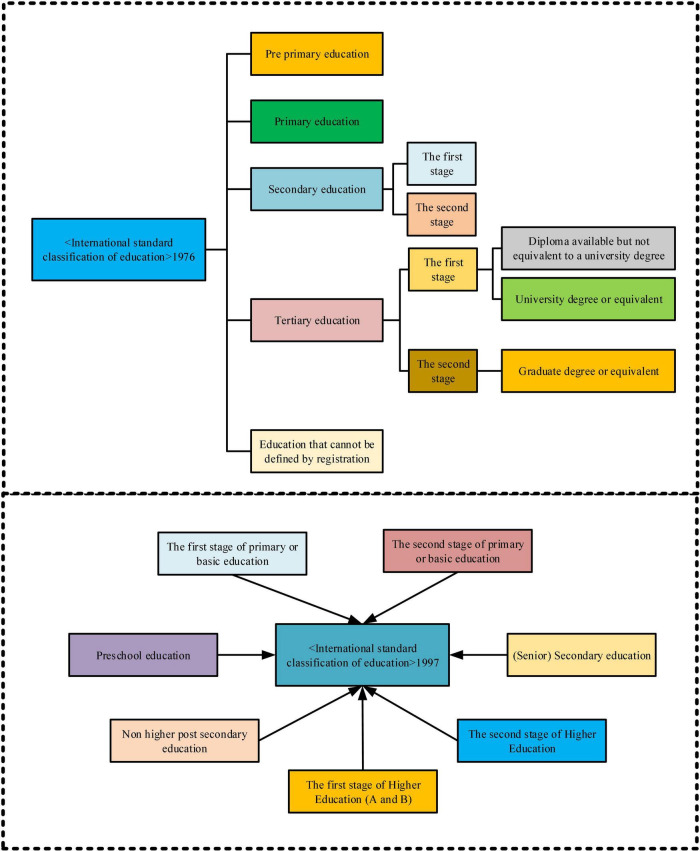
International standard classification of education.

In October 1998, UNESCO held the first World Higher Education Conference in Paris, France. The conference published the *World Higher Education Sayings* (Maria et al., 2019). Among them, *Higher Education in the 21st Century: Prospects and Actions in the World* has made a new definition of higher education, that is, adopted the *Recommendations on Recognition of Higher Education Qualifications and Qualifications* approved by the 27th session of the UNESCO General Conference in 1993. “In the definition (Karaali and Vacher, 2020), higher education includes all types of post-secondary education, training or research training approved by universities or other educational institutions of higher education institutions (Pominova and Zaretskaya, 2019). In addition to highlighting the advanced nature of higher education, this definition also uses the broadest possible definition to reflect its tolerance to various situations in countries and regions around the world (Anton et al., 2019). This is in line with the purpose of UNESCO and is consistent with its consistent position (Vladislav et al., 2019). The specific situation is shown in Figure 1.

Nowadays, China’s higher education has entered a strategic period of shifting from the extensional development of scale expansion to focusing on quality and content. Meanwhile, China’s higher education crisis and opportunities coexist. A significant issue facing higher education policymakers is steadily advancing innovation strategies, diversified development strategies, international development strategies, and market-oriented development strategies (Aleksandr and Olga, 2019).

#### Talent Training Theory

**FIGURE 2:**
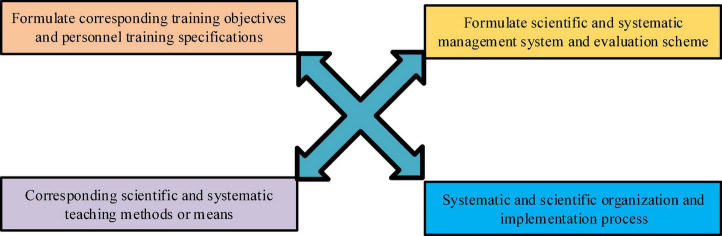
Talent training model module.

The talent training model is on educational thought and theory as the guiding ideology, and the corresponding management system and a series of evaluation plans are formulated to achieve the training goals. It adopts corresponding, scientific, and systematic education and teaching content and curriculum system to carry out scientific and systematic education (Haverkamp et al., 2018; Couperus, 2019). The concrete module is shown as in Figure 2.

Figure 2 can be simplified into: target process and method (teaching content, curriculum management, and evaluation system teaching methods). This involves many elements such as training goals, professional settings, curriculum system, and education evaluation. It includes multiple links such as setting goals, implementing the training process, evaluating, and improving training (Zolotykh et al., 2019).

### Assumptions of New Ventures Statistics

The development of new ventures cannot be separated from technological innovation, and technological innovation cannot be separated from the power of talents. As the source of enterprise technological innovation, innovative talents, whether they can be realized or not depends on whether the enterprise can effectively realize technological innovation. This leads to the assumptions in Table 1.

**TABLE 1:** Assumptions of new ventures statistics.

H1	There is a positive correlation between the talent elements of new ventures and the innovation performance of enterprises.
H2	There is a positive correlation between the R&D investment of new ventures and the innovation performance of the enterprise.
H3	There is a positive correlation between the talent elements of new ventures and R&D investment.
H4	The influence of talent elements of new ventures on enterprise innovation performance is higher than that of R&D investment.

Table 1 shows that the relationship among enterprise innovation performance, R&D investment, talent elements, and employee education level are used as the main research indicators. The subsequent research content is also mainly carried out around the above assumptions.

### Data Collection and Analysis Methods

“*Statistics of New Ventures in City S in 2019*” was used for empirical analysis.

First, the relevant data in the table is taken logarithmically. The logarithmic function is a monotonically increasing function in its domain, so taking the logarithm will not change the nature and correlation of the data. The scale of the variables is compressed, making the data more stable, and the collinearity and heteroscedasticity of the data are also weakened.

Then, the talent capital elements in the table are divided into three categories: employee size, employee qualifications (technical title), and employee education. Next, each category is subjected to factor analysis separately. After the standardization of human capital elements, R&D investment, and innovation performance is completed, the correlation is analyzed. The Pearson coefficient is used as an indicator for correlation analysis, and the strength of the influence of one variable on another variable or the closeness of the relationship between the variables is studied. The correlation coefficient r is a standardized score, and its value is not affected by the characteristics of the variable. The higher the absolute value of the correlation coefficient, the higher the degree of correlation between variables. After the correlation analysis is completed, multiple regression analysis is used to test whether there is a linear dependence between the elements of human capital, R&D investment, and innovation performance. The enter regression method is analyzed, and the contribution degree of each variable to the dependent variable is tried to be obtained. SPSS22.0 software is used for statistical processing of the data.

### Measurement and Evaluation Methods of Each Element

The content of the indicators for measurement and evaluation is shown in Table 2.

**TABLE 2:** Selecting the index classification of measurement and evaluation.

First-level indicators	Secondary indicators
Number of employees	Total number of employees at the end of the year (X1)
	Personnel engaged in scientific and technological activities (X2)
	Full-time staff (X3)
Staff qualifications	Senior technical title personnel (X4)
	Intermediate technical title personnel (X5)
Educational level of employees	Doctoral degree personnel (X6)
	Master degree personnel (X7)
	Bachelor degree holders (X8)
Corporate innovation performance	Number of patent applications (X9)
	Number of invention patent applications (X10)
	Number of patents granted (X11)
	Number of invention patents granted (X12)
	High-tech products (services) (X13)
R&D investment	Expenditures for scientific and technological activities within the enterprise (X14)
	Formed fixed assets for scientific and technological activities in the year (X15)
	Use funds from government departments for scientific and technological activities (X16)
	Expenditures for entrusting external units to carry out scientific and technological activities (X17)

*X1–X17 are all the variables after the logarithm of the original variable and the standard normal transformation. In order to analyze the relationship between employee size, employee qualifications, employee education, R&D investment, and innovation performance, this research experiment first conducts principal component analysis (PCA) on each element.*

### Analysis Sample of the Correlation Between Talent Capital Elements and Innovation Performance

**FIGURE 3:**
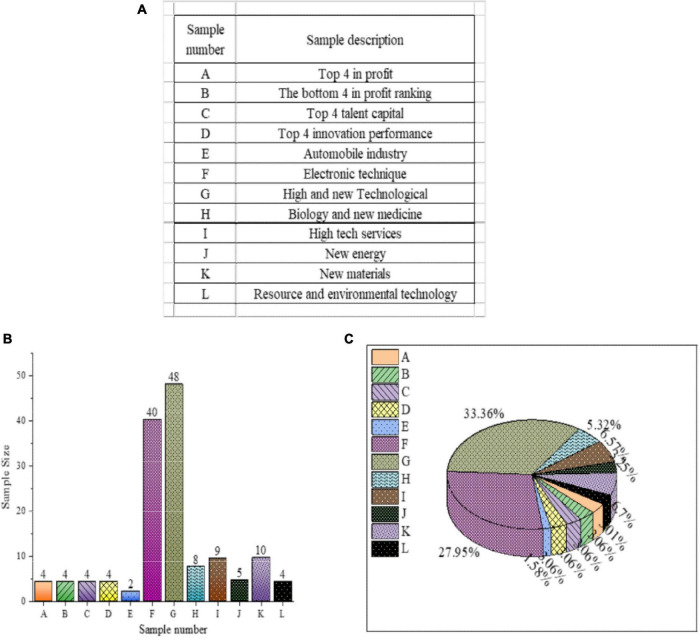
Sample situation: **(A)** Sample description; **(B)** Sample size; **(C)** Proportion of sample.

After SPSS22.0 software is used to measure and analyze the above indicators, the standardized scores of employee size S, employee qualifications Z, employee education level E, talent capital H, R&D investment R and innovation performance C are obtained. The bivariate method was adopted for Pearson correlation analysis. Before performing correlation analysis, data samples need to be classified, different samples are tested for correlation, and the differences and common points are found. Figure 3 presents the sample classification.

In Figure 3, A–D are the samples selected according to the standardized score sorting. E–L is classified according to the industry category of statistical data. Due to the incomplete information of some companies, the total number of companies classified by industry category is less than the total number of samples. The top four companies in the talent capital ranking do not take samples because most of the various personnel data in the table are 0 and cannot be processed. The bottom four companies in the ranking of innovation performance also did not draw samples, and no conclusion could be drawn during correlation analysis, so the samples were discarded.

### Data Analysis and Test Results

#### Test Results of the First-Level Hypothesis Index and the Second-Level Hypothesis Index

**FIGURE 4:**
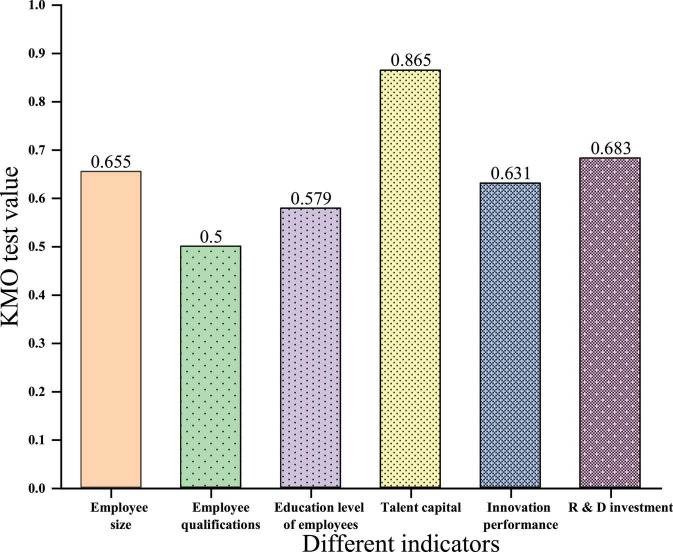
Kaiser–Meyer–Olkin test value in first-level indicators.

First, the Kaiser–Meyer–Olkin (KMO) test of various primary indicators the result is shown in Figure 4.

**FIGURE 5:**
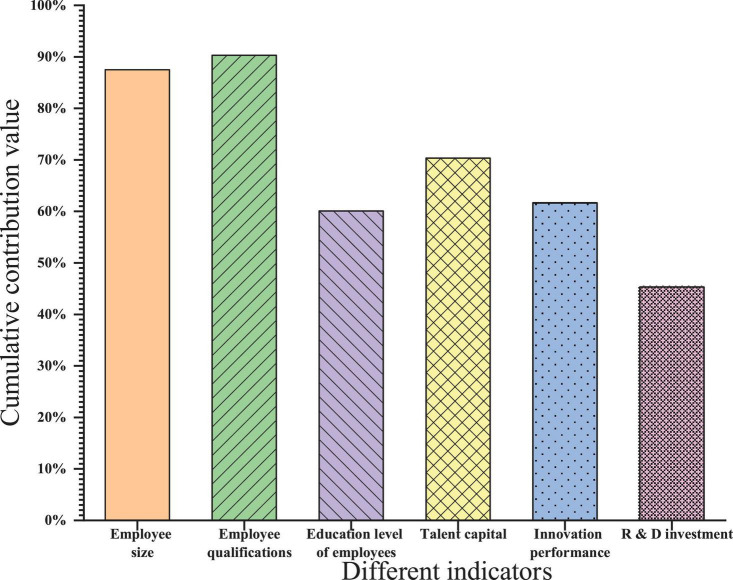
Cumulative contribution value of variance in the first-level indicator.

The effect is best because the KMO test value is greater than 0.9. A test value of 0.7–0.9 is acceptable. When the test value is less than 0.5, it means it is not suitable, and factor analysis is required. Figure 4 shows that it is acceptable when the KMO test value of the employee size is 0.655. When the KMO test value of employee qualifications are 0.5, it is reluctant to accept it. When the KMO test value of an employee’s education level is 0.579, it is reluctant to accept it. When the KMO test value of human capital is 0.865, it is very suitable. When the KMO test value of innovation performance is 0.631, it is reluctant to accept it. When the KMO inspection value of R&D investment is 0.683, it is reluctant to accept it. Therefore, factor analysis can be performed on staff size, qualifications, education, talent capital, innovation performance, and R&D investment. The cumulative contribution value of the variance of each first-level indicator is shown in Figure 5.

In order to facilitate the correlation analysis for follow-up research, the PCA method is adopted. The principle of extracting the number of principal components is: the feature value is greater than 1 and the cumulative contribution value of the component is ≥85%. In Figure 5, the cumulative contribution value of employee size is 87.496%. The obtained common factor is denoted as S. The cumulative contribution value of employee qualifications is 90.309%. The obtained public factor is recorded as Z. The cumulative contribution value of the employee’s education level is 69.079%, and the obtained public factor is denoted as E. The cumulative contribution value of human capital is 70.322%, and the obtained public factor is recorded as H. The cumulative contribution value of innovation performance is 61.658%, and the obtained public factor is recorded as C. The cumulative contribution value of R&D investment is 45.306%, which is very low, indicating that the performance of this common factor is approximately general data. But a common factor is still obtained, denoted as R.

**FIGURE 6:**
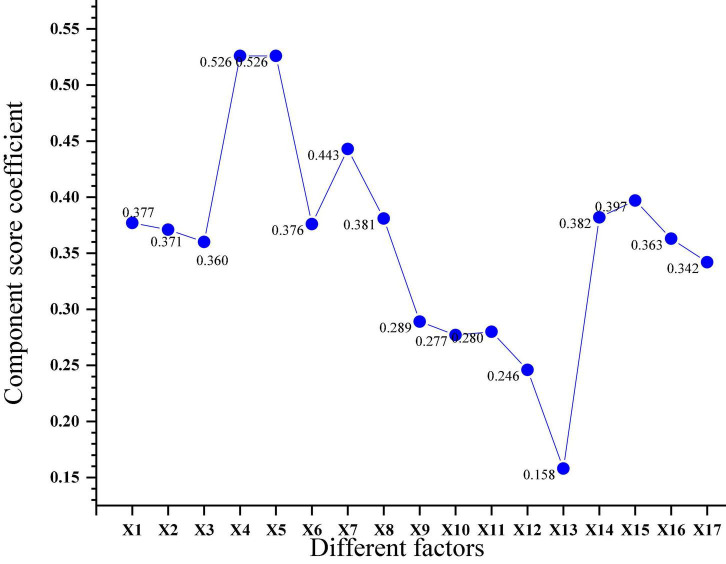
Score coefficients of secondary index components.

Next, the secondary indicators X1–X17 are subjected to PCA. The results obtained after the analysis are shown in Figure 6.

The component score coefficients X1–X17 in Figure 6, combined with the public factors obtained from the above analysis, the factor scores function of employee size, employee qualifications, employee education, talent capital, innovation performance, and R&D investment can be combined be calculated. The specific function is shown in Table 3.

**TABLE 3:** Factor score function in the first-level indicators.

First level indicator	Factor score function
Number of employees	*S* = 0.337*X*1 + 0.371*X*2 + 0.360*X*3 (1)
Staff qualifications	*Z* = 0.526*X*4 + 0.526*X*5 (2)
Educational level of employees	*E* = 0.376*X*6 + 0.443*X*7 + 0.381*X*8 (3)
Talent capital	*H* = 0.149*X*1 + 0.167*X*2 + 0.161*X*3 + 0.140*X*4 + 0.149*X*5 + 0.116*X*6 + 0.156*X*7 + 0.149*X*8 (4)
Corporate innovation performance	*C* = 0.289*X*9 + 0.277*X*10 + 0.280*X*11 + 0.246*X*12 + 0.158*X*13 (5)
R&D investment	*R* = 0.382*X*14 + 0.397*X*15 + 0.363*X*16 + 0.342*X*17 (6)
	

#### Correlation Analysis Data of Talent Capital Elements and Innovation Performance

**FIGURE 7:**
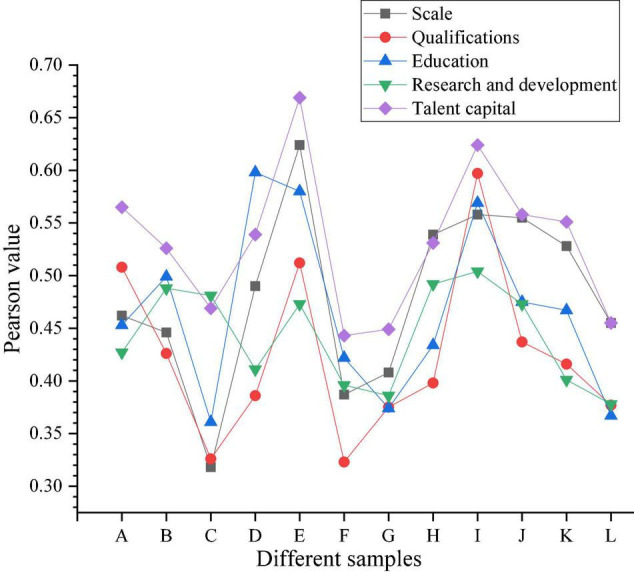
Correlation analysis of Pearson coefficient (innovation performance).

The experiment performed Pearson correlation analysis on the classified samples, and obtained the correlation coefficients of innovation performance and other factors in each sample, as shown in Figure 7:

The Pearson value of all the coefficients in Figure 7 is 0, significantly correlated at the 0.01 level (two-sided). The coefficients are all positive, indicating a positive effect. The closer the coefficient is to 1, the higher the degree of positive correlation. In Figure 7, the coefficient of the overall sample size is 0.509, indicating a moderately positive correlation between the employee size of the entire sample and the innovation performance of the enterprise. The coefficient of employee qualifications is 0.452, indicating a positive correlation between qualifications and innovation performance in the sample. The highest value of the correlation coefficient of education is 0.598, indicating that among the top 4 innovation performance companies, the education level of employees has the highest correlation to the innovation performance of the company. In research and development, the highest correlation coefficient appears in the high-tech service industry, with a coefficient of 0.504. Among talent capital, the coefficient of the automobile industry is the highest at 0.669.

**FIGURE 8:**
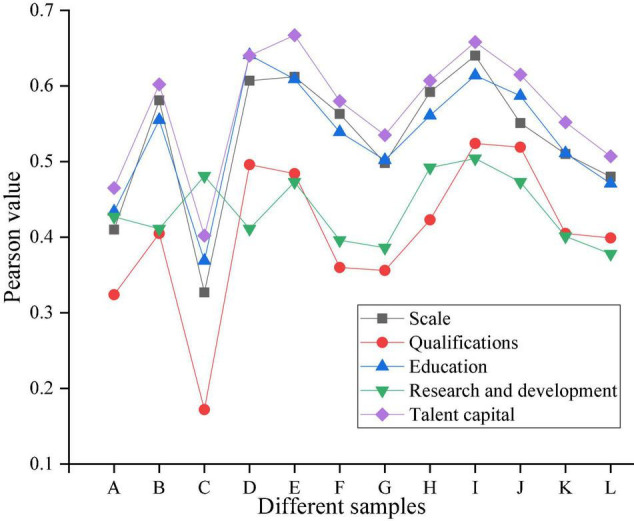
Correlation analysis of Pearson coefficient (R&D investment).

The correlation coefficient between R&D investment and each item is shown in Figure 8.

According to Figure 8, it is necessary to verify the correlation between R&D investment and human capital elements to be analyzed. All *P* = 0 is significantly correlated at the 0.01 level (two-sided), and the coefficients are all positive, indicating that there is a positive influence between them. Among them, the highest coefficients of scale and qualifications come from the high-tech service industry, reflecting the relatively high correlation between the scale of employees in this industry, employee qualifications, and corporate R&D investment. Education Among the innovation samples ranked by innovation performance, the correlation coefficient of employee education level is the highest, and the correlation coefficients of the rest of the human capital elements are also quite close (Deng et al., 2021). This shows that there is a moderate positive correlation between employee education and R&D investment for companies with outstanding innovation performance.

Therefore, combined with the relevant data in Figures 7, 8, the second part of the hypothesis has been verified:

H1: There is a positive correlation between the talent capital elements of a new venture enterprise and the innovation performance of the enterprise, and it is established.

H2: The R&D investment of new ventures is positively correlated with the company’s innovation performance established.

H3: There is a positive correlation between the R&D investment of new ventures and the elements of human capital, and it is established.

H4: The impact of the talent capital element of a new venture on the innovation performance of the enterprise is higher than the investment in R&D, established.

H5: In the sub-items of human capital elements of new ventures, the education level of employees has the greatest impact on the innovation performance of the enterprise, and it is only established in certain industries and companies with outstanding innovation performance.

### The Practical Framework for Training Innovative Talents

**FIGURE 9:**
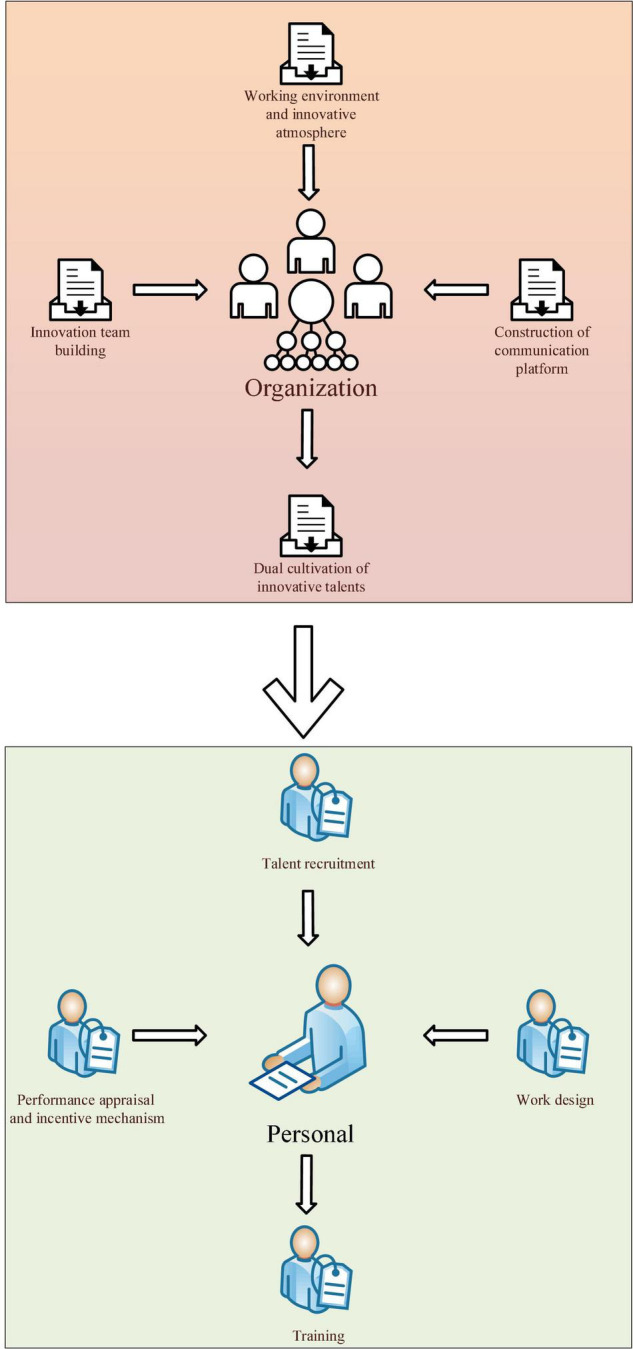
The practical framework for training innovative talents.

Through the case analysis of the above two companies, the proposed method summarizes the practice of training innovative talents into the framework in Figure 9.

Firstly, at the organizational level: (1) The construction of an innovation team should be highly valued, and the team should promote innovation as a unit. Team members cooperate by common goals in project management, forming a benign interactive relationship. This immersive training mechanism can permeate everyone through the organizational innovation atmosphere, and can quickly convey the awareness of innovation in the project team, and promote everyone to actively participate in innovation. (2) The working environment and innovation atmosphere. Innovative talents are intellectually intensive people. The management of such talents cannot use traditional rules and regulations. An open and relaxed environment must be provided to help them relax their emotions and generate innovative inspiration more easily. (3) The construction of an innovative interactive platform. The difference between this platform and the innovation team is that it contains more cross-border experts, obtains a wider range of technical support, gets more attention from the top of the enterprise, and has more opportunities to obtain organizational financial support. (4) The dual training of innovative talents. Because the company adopts the innovation team model, there will be two training institutions for employees: a functional department and an innovation project team.

Secondly, on the personal level: (1) Talent recruitment. The more highly qualified and high-level talents are, the faster they can master the knowledge, experience, and skills after receiving training. Therefore, technology companies need to pay attention to the basic qualities of employees, such as academic qualifications, experience, vocational skills, etc., when recruiting talents. This infers its innovation potential. For some rare technical majors, companies can directly establish cooperation with universities and their affiliated research institutions, accept recommendations from universities, entrust universities to train specific professionals, or even provide internship opportunities for designated professional students to train potential employees. (2) Work design. Work design for employee positions is the design of work content, work functions and work relationships to achieve organizational goals and meet individual needs effectively. Work design consists of work content, work functions, work relationships, work performance, and result feedback. In the management of innovative talents, companies should design the positions of their employees from the start of their employment. A detailed job design enables employees to realize that the company attaches importance to themselves and enables employees to quickly grasp the company’s job expectations. (3) Training is the most direct and effective means for companies to enhance their talents’ innovation capabilities. Enterprises should design a complete multi-level training system. (4) Performance appraisal and incentive mechanism. Short-term performance appraisal is required when an employee is in an innovation team and takes the project as the goal. The progress and quality of the project are assessed on a monthly and quarterly basis. The appraiser is often the team leader. Through short-term assessment, employees can clearly recognize the value of their contribution to the team, clarify follow-up goals, and work hard toward realizing team goals more accurately. The long-term appraisal is an annual appraisal of employees carried out by the leaders of functional departments. From the difference of appraisers, the characteristics of dual management of innovative talents can also be seen.

## Discussion

### Case Discussion

From the perspective of empirical analysis, the purpose is to draw conclusion that are instructive in the practice of training innovative talents. Therefore, the case analysis is carried out in combination with specific enterprises to find the basis of the empirical conclusion. SQ Group and ZX International were selected as the main case study objects. The factor function score rankings of the two companies are shown in Table 4.

**TABLE 4:** The score function of the factors in the first-level indicators.

Company name	SQ group	ZX international
Industry category	vehicle	electronic
Is it a high-tech enterprise	Yes	Yes
Innovation performance score ranking	11	1
Profit score ranking	1	21
Talent capital score ranking	3	15
R&D investment score ranking	2	65
Employee size score ranking	3	22
Employee education level score ranking	2	6
Employee qualification score ranking	11	123

Table 4 shows that the innovative talent management strategies embodied by companies engaged in different industries will be different, each with its own focus. Through the case study of SQ Group, SQ Group focuses on the management of innovative talents to provide employees with adequate external protection. For example, a comprehensive innovation material incentive mechanism and the construction of an innovation platform. The guarantee of external conditions can ensure that employees can innovate without any worries. Such measures are more suitable for talents who have the willingness to innovate and can innovate. For such employees, companies do not need to emphasize training on their knowledge and skills, nor do they need to use various methods to stimulate their enthusiasm for innovation. They already possess the basic qualities of independent innovation. Therefore, the use of external safeguards can promote effective innovation of such employees. Of course, SQ Group also has related measures to build a team of innovative talents. The two-pronged approach of external introduction and internal training makes the scale of innovative talents sufficient to promote the overall technological innovation of the enterprise.

In the case study of ZX International, ZX International pays more attention to the spiritual protection of employees. Firstly, adequate spiritual incentives. The lunch meeting between employees and business leaders or public praise is a kind of satisfaction to the needs of employees to realize their self-worth (Feng et al., 2021a). Secondly, ZX International pays attention to the construction of an innovative atmosphere. It is to subtly implant an innovation culture in the employees so that the seeds of innovation can take root in the hearts of employees. Finally, ZX International also emphasizes the sense of belonging of employees to the company and an open communication environment. This is a kind of satisfaction to the needs of employees at the spiritual level. These spiritual guarantees are designed to stimulate every employee’s sense of innovation, making them proactive and willing to innovate. If the employees’ motivation is not sufficient, ZX International has also adopted the auxiliary means of performance appraisal, using external forces to promote and encourage employees to innovate.

The difference in the focus of innovative talent management practices between the two companies stems from industry differences. For the automobile manufacturing industry, the threshold for patent-related technological innovation is relatively high, and companies need to focus on providing convenience for those who can implement innovation. In the integrated circuit industry where ZX International is located, the threshold for innovation in software development and integrated circuit design is much lower than that of the automobile manufacturing industry. The innovation ability of employees is similar, and the key lies in whether employees are willing to innovate. Therefore, the enterprise’s talent management focuses on the work of stimulating employees’ sense of innovation and enhancing the enterprise’s innovative atmosphere.

## Conclusion

With China’s industrial upgrading and economic structure adjustment, the shortage of medium and high-quality technical and skilled personnel is becoming more and more serious. Based on technological innovation, talent capital, and innovative talent management theories, this study proposes a hypothesis of the relationship between talent capital, R&D investment, and enterprise innovation performance. In order to verify these hypotheses, by the statistical data of new ventures that are now available, statistical analysis methods are used to analyze the hypotheses to prove the validity empirically (Feng et al., 2021b).

After PCA and correlation analysis, the initial hypothesis is confirmed. These conclusion have been drawn: talent capital has a positive correlation to corporate innovation performance; R&D investment has a positive correlation to corporate innovation performance; and R&D investment positively correlates to human capital. After further regression analysis, it is concluded that the influence of talent capital elements on enterprise innovation performance is higher than that of R&D investment. In some specific cases, the education level of employees has the greatest impact on innovation performance. The framework of innovative talent management practices is summarized. Discussing practical and feasible talent management strategies from the organizational and individual levels can play a certain guiding role in the actual management of technology companies.

The deficiencies are: (1) in the data analysis of this experiment, the small sample size leads to a higher degree of convergence, resulting in a low comprehensiveness of the analysis conclusion; (2) due to limited space, fewer research objects are selected for analysis. After that, the lower-ranked companies need to be further studied.

## Data Availability Statement

The raw data supporting the conclusions of this article will be made available by the authors, without undue reservation.

## Ethics Statement

The studies involving human participants were reviewed and approved by Hunan University of Humanities, Science and Technology Ethics Committee. The patients/participants provided their written informed consent to participate in this study. Written informed consent was obtained from the individual(s) for the publication of any potentially identifiable images or data included in this article.

## Author Contributions

All authors listed have made a substantial, direct, and intellectual contribution to the work, and approved it for publication.

## Conflict of Interest

The authors declare that the research was conducted in the absence of any commercial or financial relationships that could be construed as a potential conflict of interest.

## Publisher’s Note

All claims expressed in this article are solely those of the authors and do not necessarily represent those of their affiliated organizations, or those of the publisher, the editors and the reviewers. Any product that may be evaluated in this article, or claim that may be made by its manufacturer, is not guaranteed or endorsed by the publisher.
